# Microbiome and intestinal pathophysiology in post-acute sequelae of COVID-19

**DOI:** 10.1016/j.gendis.2023.03.034

**Published:** 2023-06-19

**Authors:** Jilei Zhang, Yongguo Zhang, Yinglin Xia, Jun Sun

**Affiliations:** a *Division of Gastroenterology and Hepatology*, *Department of Medicine*, *University of Illinois Chicago*, *IL* 60612, *USA*; b *UIC Cancer Center*, *Department of Microbiology and Immunology*, *University of Illinois Chicago*, *Chicago*, *IL* 60612, *USA*; c *Jesse Brown VA Medical Center*, *Chicago*, *IL* 60612, *USA*

**Keywords:** Gastrointestinal symptoms, GI tract, Gut microbiome, Inflammatory bowel diseases, Intestinal barrier, Long COVID, Metabolites, Tight junctions

## Abstract

Long COVID, also known for post-acute sequelae of COVID-19, describes the people who have the signs and symptoms that continue or develop after the acute COVID-19 phase. Long COVID patients suffer from an inflammation or host responses towards the virus approximately 4 weeks after initial infection with the SARS CoV-2 virus and continue for an uncharacterized duration. Anyone infected with COVID-19 before could experience long-COVID conditions, including the patients who were infected with SARS CoV-2 virus confirmed by tests and those who never knew they had an infection early. People with long COVID may experience health problems from different types and combinations of symptoms over time, such as fatigue, dyspnea, cognitive impairments, and gastrointestinal (GI) symptoms (*e.g.*, nausea, vomiting, diarrhea, decreased or loss of appetite, abdominal pain, and dysgeusia). The critical role of the microbiome in these GI symptoms and long COVID were reported in clinical patients and experimental models. Here, we provide an overall view of the critical role of the GI tract and microbiome in the development of long COVID, including the clinical GI symptoms in patients, dysbiosis, viral–microbiome interactions, barrier function, and inflammatory bowel disease patients with long COVID. We highlight the potential mechanisms and possible treatment based on GI health and microbiome. Finally, we discuss challenges and future direction in the long COVID clinic and research.

## Introduction

**Figure 1:**
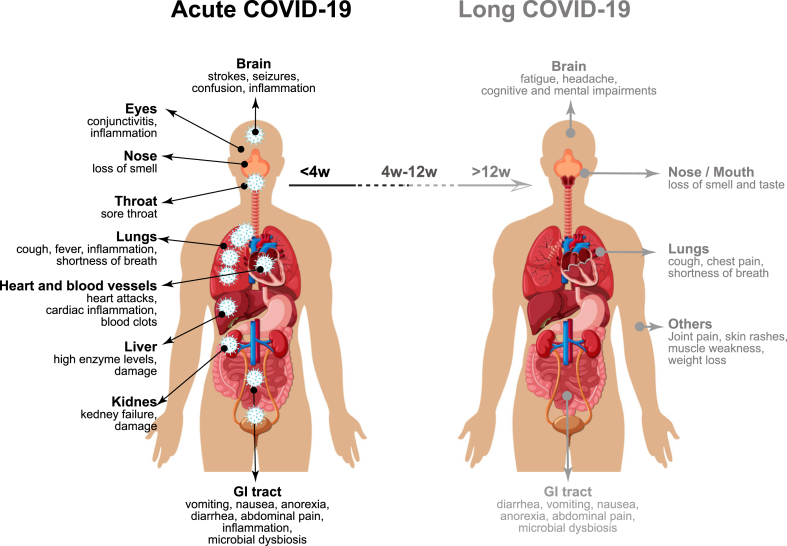
Multiple symptoms of acute COVID-19 remained in long COVID. In acute COVID-19 patients, the SARS-CoV-2 virus is grounded in the lungs but can extend to many organs, including the heart and blood vessels, kidneys, brain, liver, and gastrointestinal tract. Multiple symptoms lasted more than 12 weeks in long COVID patients, except fatigue, the most common symptom in long COVID reports.

In the past 2.5 years, the coronavirus diseases 19 (COVID-19) pandemic, which is induced by severe acute respiratory syndrome coronavirus 2 (SARS-CoV-2), has resulted in millions of infections and deaths and a major strain on health systems worldwide. Disorders of multisystem due to SARS-CoV-2 infection, or acute COVID-19, have been well described (Fig. 1). Patients report various symptoms after recovery from acute COVID-19. The increasing number of reports of symptoms post-COVID-19 infection or long COVID has attracted the attention of scientists and health workers.

The term long COVID describes the people who have the signs and symptoms that continue or develop after the acute COVID-19 phase and should include both ongoing symptomatic COVID-19 and post-COVID-19 syndrome.^1^^,^^2^ People with long COVID may experience health problems from different types and combinations of symptoms over different lengths of time. The most typical signs and symptoms of long COVID are fatigue, dyspnea, cognitive impairments, and gastrointestinal (GI) disorders.^1^^,^^3^, ^4^, ^5^, ^6^ Other reported symptoms include mood changes, anxiety, insomnia, headache, sore throat, smell and taste dysfunctions, cough, chest pain, palpitations, tachycardia, myalgia, joint pain, hair loss, and skin rashes^7^^,^^8^ (Fig. 1). These symptoms seem frequent and do not affect only those patients who have experienced the most severe forms of acute COVID-19. The considerable number of patients affected by long COVID highlights the importance of this topic because these symptoms frequently exert a substantial effect on patients' health and life quality.

However, long COVID is still relatively unknown. What is long COVID, and how to diagnose it? What is the role of the GI tract in long COVID? Are there any potential therapies for long COVID based on the GI tract? This synthetic review seeks to answer these questions.

## Long COVID

Long COVID describes the people who have the signs and symptoms that continue or develop after the acute COVID-19 phase and should include both ongoing symptomatic COVID-19 and post-COVID-19 syndrome.^1^^,^^2^ The term “long-COVID-19” was first used by patient Elisa Perego from Italy as a hashtag on her social media in May 2020 to describe her experience of continuing symptoms of COVID-19 even after recovery.^2^ After that, it was moved through various social media to formal clinical experts and policymakers and gained reasonable consideration for these patients. Recently, COVID-19 infections have been divided into three phases, acute COVID-19 with signs and symptoms of COVID-19 infection for up to 4 weeks, ongoing symptomatic COVID-19 with signs and symptoms from 4 weeks to 12 weeks, and post-COVID-19 syndrome with signs and symptoms for more than 12 weeks, according to the joint guidelines proposed by the National Institute for Health and Care Excellence (NICE), the Scottish Intercollegiate Guidelines Network (SIGN), and the Royal College of General Practitioners (RCGP).^1^^,^^2^ Nowadays, long COVID, also known for post-acute sequelae of COVID-19 ^9^, post-COVID conditions, post-acute COVID-19, long-term effects of COVID-19, chronic COVID-19, or long-haul COVID, is defined as the condition of the people who suffer with an inflammation or host responses towards virus approximately 4 weeks after initial infection with the SARS CoV-2 virus which continues for an uncharacterized duration.^3^, ^4^, ^5^ Anyone infected with COVID-19 before could experience long-COVID conditions, including the patients who were infected with SARS CoV-2 virus confirmed by tests and those who never knew they had an infection early.

People with long COVID may experience health problems from different types and combinations of symptoms over different lengths of time. The most typical signs and symptoms of long COVID are fatigue, dyspnea, and cognitive impairments.^1^^,^^6^, ^7^, ^8^ Other reported symptoms include mood changes, anxiety, insomnia, headache, sore throat, smell and taste dysfunctions, cough, chest pain, palpitations, tachycardia, myalgia, joint pain, hair loss, and skin rashes.^8^^,^^10^ More importantly, the GI symptoms in patients with acute COVID-19 could be persistent in the long COVID, such as nausea, vomiting, diarrhea, decreased or loss of appetite, abdominal pain, and dysgeusia.^8^^,^^10^, ^11^, ^12^ The critical role of the intestinal microbiome in these GI symptoms and long COVID were also reported in clinical patients or animal models.^12^, ^13^, ^14^

## GI pathobiology in the long COVID

It is widely known that the SARS-CoV-2 infection mainly affects the respiratory system, but various other organs/tissues can also be affected with many different unknown outcomes.^10^^,^^15^^,^^16^ Similar to acute COVID-19 causing GI problems, such as diarrhea,^17^, ^18^, ^19^ recent evidence suggests that the GI symptoms could be persistent in long COVID patients^10^^,^^20^^,^^21^ (Table 1). For instance, it is reported that 87.4% of patients who had recovered from COVID-19 showed persistence of at least one symptom, including GI symptoms.^21^ These GI symptoms include nausea, vomiting, abdominal pain, dysgeusia/ageusia, anorexia, lack/loss of appetite, and diarrhea (Table 1). Diarrhea is the most common problem reported in almost all the long COVID studies with variable percentages (0%–58.7%), which largely depends on the cohort size of the study. Meanwhile, some of the studies, especially the ones with a large volume of patients, were based on a questionnaire that was entirely based on patients' self-report, which could lose some information or be missed with other diseases with similar symptoms, such as food poisoning or inflammatory bowel diseases (IBDs).

**Table 1:** Summary of demographic and GI symptoms from the studies of long COVID subjects.

Study	Country	Number of subjects	Age range, mean (Years)	Female/Male	Body mass index (mean)	Assessed time (days)	Nausea	Vomiting	Diarrhea	Abdominal pain	Dysgeusia or Ageusia	Anorexia	Lack/loss of appetite
Carfi A et al^21^	Italy	143	19–84, 56.5	53/90	26.3	60.3	Not reported (NR)	NR	2.8%	NR	8.5%	NR	8.3%
Goërtz YMJ et al^109^	Netherlands and Belgium	2113	39–54, 47	1803/310	25.2	79	36.5%	9.0%	41.1%	NR	42.3%	NR	NR
Garrigues E et al^110^	French	120	NR, 63.2	45/75	29.2% < 25;	110.9	NR	NR	24.2%	NR	10.8%	NR	NR
					47.5% ≥ 25								
					23.3% missing								
Xiong Q et al^111^	China	538	22–79, 52.0	293/245	NR	97	4.1%		10.2%	NR	NR	NR	NR
Dennis A et al^112^	United Kingdom	201	21–71, 44.0	142/59	25.7	141	NR	NR	58.7%	53.7%	NR	NR	NR
Carvalho-Sneider C et al^113^	French	130	30^−^–80^+^, 49	68/62	NR	60	NR	NR	30.8%	NR	60.0%	NR	NR
Davis HE et al^114^	56 countries	3762	18–80^+^, NR	2969/718	NR	>180	19.9%	2.9%	20.5%	19.2%	25.2%	NR	13.7%
Cellai M et al^115^	USA	26	23–78, 47.5	20/6	53.8% > 30	42	19.2%	NR	11.5%	3.8%	NR	11.5	NR
Wang X et al^20^	China	131	18–88, 49	72/59	NR	21–28	0.76%	0	0	NR	NR	NR	NR
Daher A et al^116^	Germany	33	NR, 64	11/22	NR	48–71	6%	NR	9%	3%	9%	NR	NR
Huang C et al^117^	China	1655	NR, 57	199/856	NR	153	NR	4.8%		NR	7.3%	NR	8.3%
Tenforde MW et al^118^	USA	270	18–50^+^, NR	140/130	NR	14–21	13%	NR	14%	18%	28%	NR	NR
Eiros R et al^119^	Spain	139	NR, 52	100/39	NR	72.8	NR	NR	NR	4%	5%	NR	NR
Weng J et al^120^	China	117	<60, 45.3%;	52/65	NR	90	18%	<10%	15%	14%	NR	NR	24%
			≥60, 44.7%										
Salvatori S et al^56^	Italy	21	21–68, 43	14/7	NR	>28	NR	NR	14%	NR	NR	NR	NR
Zollner A et al^57^	Austria	46	NR, 44.7	20/26	23.0	219	NR	NR	15.2%	21.7%	NR	NR	NR
Shah SC et al^11^	USA	218,045	18–100, 59.2	33,345/184,710	NR	>60	7.%		9.8%	NR	NR	NR	NR
Fernández-de-Las-Peñas C et al^121^	Spain	1969	NR, 61	915/10,54	NR	240	NR	NR	2.5%	NR	2.7%	NR	NR

Liver diseases are the other GI diseases with high morbidity and mortality, such as nonalcoholic fatty liver disease (NAFLD), which has been classically described as a barometer of metabolic health and carries a high risk of cardiovascular complications and mortality.^22^ Metabolic-associated fatty liver diseases were reported as highly prevalent after the hospital discharge of patients with long COVID.^23^ Even though long-term outcomes in patients with liver dysfunction in acute COVID-19 are sparse, signs of fibro-inflammation in 5 out of 52 patients were found by performing liver MRI (magnetic resonance imaging) at 2–3 months after disease onset.^24^ The findings that a short-term increase in alcohol consumption during the COVID-19 pandemic could substantially increase long-term alcohol-associated liver disease-related morbidity and mortality highlight the need for individuals and policymakers to make informed decisions to mitigate the impact of high-risk alcohol drinking in the post-pandemic era.^25^ As one of the wide-used therapy for liver diseases, liver transplant (LT) is facing an increasing burden during and post-COVID-19 pandemic in redesigning service provision, restructuring outpatient care, selecting donors and recipients, and performing LT surgery. This could further negatively impact the care of patients with decompensated cirrhosis who require ongoing medical attention and potential LT and care of patients after LT.^26^ It is reported that LT recipients show a lower long-term persistence of anti-SARS-CoV-2 antibodies compared with non-transplanted patients.^9^

## GI microbiome and metabolites in long COVID

It is well known that microbiota widely colonizes our body and varies between individuals and ethnicities.^27^ Even though the role and mechanisms of the microbiota have not been fully elucidated, the microbiome, especially the intestinal microbiome, has been currently considered to be associated with lots of human diseases in both GI tract, such as IBDs,^28^ colorectal cancer,^29^ and type 2 diabetes,^30^ and beyond, such as Parkinson's disease,^31^ and amyotrophic lateral sclerosis.^32^ Moreover, respiratory infections are directly or indirectly associated with intestinal microbiota patterns via the gut–lung axis.^33^^,^^34^ Similarly, the critical role of the intestinal microbiome in both acute SARS-CoV-2 infections and long COVID has been widely reported, although the mechanisms behind it are still unclear.^12^, ^13^, ^14^^,^^17^^,^^35^, ^36^, ^37^ One of the possibilities is through the intestinal epithelium for their importance in balancing the microbial community, as previously reported that gut microbiome dysbiosis induced by SARS-CoV-2 in mice was correlated with alterations to Paneth cells and goblet cells and markers of barrier permeability.^37^ The group from Hong Kong reported that gut dysbiosis could persist for six months and one year in patients with long COVID with significantly lower bacteria diversity and richness and distinctly separated beta diversity.^36^^,^^38^ These gut microbiome dysbiosis could further cause life-threatening secondary infections, such as the translocation of bacteria from the intestine into the systemic circulation in COVID-19 patients, which was supported by analyzing blood culture results in testing for secondary microbial bloodstream infections with paired microbiome data.^37^ Using tongue swabs from patients persisting with COVID-19 symptoms, Haran et al reported that these patients have a significantly higher abundance of microbiota that induced inflammation, such as members of the genera *Prevotella* and *Veillonella*.^39^ This study suggests the association between the oral microbiome and long COVID, revealing the possibility that the oral microbiome's dysfunction may further impact intestine microbial homeostasis, contributing to this draining disease. The intestine is solid with microbiota and robust with metabolites from gut microbiota and the host, which may play an important role the in development, prevention, and treatment of long COVID. In a case report of a patient with long COVID with long-lasting severe GI symptoms, Wang et al found that a high intake of dietary fibers with diverse physicochemical structures could alleviate GI symptoms by shifting gut microbiota, particularly the enrichment of short-chain fatty acid-producing bacteria. This prolonged high-fiber intake might also reduce bacterial fermentation in the small intestine, which further reduces gas production and work as a potential mechanism for alleviating GI symptoms such as nausea and loss of appetite.^40^

Intestinal microbiota alterations in acute COVID-19 patients include the down-regulation of commensal bacteria from the families *Ruminococcaceae* and *Lachnospiraceae*, some of which may help strengthen the gut barrier and maintain homeostasis.^41^ *Faecalibacterium prausnitzii*, *Eubacterium rectale*, and several *Bifidobacterium* species, with potential immunomodulatory properties, were found depleted when people infected with SARS-CoV-2.^41^, ^42^, ^43^ These bacterial alterations could persist over the acute infection and even a 6-month recovery period (long COVID) after clearance of viruses.^42^^,^^44^ As part of the intestinal community, the viruses and fungi inside the gut were also altered after SARS-CoV-2 infection, such as the increase of opportunistic fungal pathogens *Candida albicans*, *Candida auris*, and *Aspergillus flavus*.^45^^,^^46^ However, whether these alterations will be persistent like the bacteria does is still unclear and more investigations need to be performed in the future. Meanwhile, as one of the viruses infecting our respiratory tract, SARS-CoV-2 infects and contacts the nasopharyngeal cells first. It is not surprising that the salivary and nasopharyngeal microbiota was altered during SARS-CoV-2 infection,^47^^,^^48^ such as depletion of oral *Bifidobacterium*, *Lactobacillus*, and *Solobacterium*, and nasopharyngeal *Paracoccus*, and enrichment of nasopharyngeal *Proteus*, *Cupravidus*, and *Lactobacillus* in severe COVID-19 patients.^47^ Similar phenomena were also found in the lower respiratory tract bacterial microbiome of COVID-19 critically ill patients who were characterized with *Pseudomonas alcaligenes*, *Clostridium hiranonis*, *Acinetobacter schindleri*, *Sphingobacterium* spp., *Acinetobacter* spp. and *Enterobacteriaceae*, while control patients characterized with lung commensal bacteria *Haemophilus influenzae*, *Veillonella dispar*, *Granulicatella* spp., *Porphyromonas* spp., and *Streptococcus* spp.^49^

**Figure 2:**
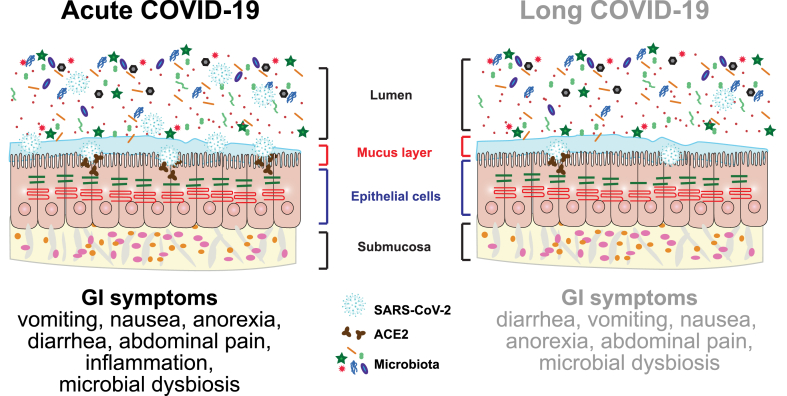
Long COVID in the intestinal tract. Like acute COVID-19, long COVID patients suffer from multiple gastrointestinal disorders, such as diarrhea, vomiting, nausea, anorexia, abdominal pain, and microbial dysbiosis. Although the SARS-CoV-2 antigen protein has been reported in long COVID patients with inflammatory bowel diseases, the virus isolation and culture failed, highlighting the possibility of virus amplification and continuous intestinal damage in long COVID patients.

Here, we summarized the most relevant studies reported in the field of gut microbiota and acute and post-acute COVID-19 (Table 2). All these findings support the emerging role of gut microbiota alterations in long COVID and the potential to be developed as a novel holistic approach for this disease by modulating microbiota (Fig. 2).

**Table 2:** The intestinal microbiota alteration in the gut of acute COVID-19 and long COVID patients.

Reference	Country	Cohort size (positive/control)	Enriched intestinal microbiota	Depleted intestinal microbiota
*Acute COVID-19*				
Sun Z et al, 2022^66^	China	63/8	p_*Verrucomicrobia*	g_*Faecalibacterium*, g_*Dialister*, g_*Lachnospira*
Newsome RC et al, 2021^122^	USA	50/34	g_*Corynebacterium*, g_*Campylobacter*, g_*Finegoldia*	g_*Klebsiella*, g_*Agathobacter*, g_*Fusicatenibacter*
Zuo T et al, 2021^46^	China	98/78	19 virus species	26 virus species
Zuo T et al, 2020^45^	China	30/30	s_*Candida albicans*, s_*Candida auris* s_*Aspergillus flavus*	None
Zuo T et al, 2020^82^	China	15/15	s_*Clostridium hathewayi*, s_*Actinomyces viscosus*, s_*Bacteroides n*ordii	s_*Faecalibacterium prausnitzii*, s_*Lachnospiraceae bacterium* 5_1_63FAA, s_*Eubacterium rectale*, s_*Ruminococcus obeum*, s_*Dorea formicigenerans*
Xu X et al, 2022^123^	China	38/31	g_*Mediterraneibacter*, g_*Blautia*, g_*Streptococcus*, g_*Anaerostipes*, g_*Anaerobutyricum*	g_*Roseburia*, g_*Klebsiella*, g_*Coprococcus*, g_*Dialister*
Romani L et al, 2022^124^	Italy	68/20	g_*Faecalibacterium*, g_*Bacteroidetes*, g_*Neisseria*	g_*Bifidobacterium*, g_*Blautia*, g_*Akkermansia*
Ren Z et al, 2021^125^	China	24/48	g_*Akkermansia*, g_*Actinomyces*, g_*Erysipelotrichaceae*, g_*Peptostreptococcaceae*, g_*Halomonas*	g_*Lachnospiraceae*, g_*Lachnospira*, g_*Megamonas*, g_*Ruminococcaceae*, g_*Faecalibacterium*
Albrich WC et al, 2022^126^	Switzerland and Ireland	130/42	g_*Bifidobacterium*, g_*Ruminococcus*, g_*Enterococcus*, g_*Eggerthella*, g_*Lachnoclostridium*, g_*Erysipelatoclostridium*, g_*Streptococcus*, g_*Flavonifractor*	g_*Faecalibacterium*, g_*Agathobacter*, g_*Dorea*, g_*Coprococcus*, g_*Lachnospiraceae*, g_*Bifidobacterium*
Schult D et al, 2022^127^	Germany	107/26	g_Proteobacteria, g_Parabacteroides, g_Lachnoclostridium	s_*Faecalibacterium prausnitzii*, s_*Blautia luti*, s_*Dorea longicatena*, s_*Gemmiger formicilis*, s_*Alistipes putredinis*, g_*Ruminococcus*, g_*Fusicatenibacter*
Yeoh YK et al, 2021^42^	China	10/68	g_*Bacteroidetes*	g_*Actinobacteria*
Chen N et al, 2020^128^	China	30/30	g_*Streptococcus*, g_*Rothia*, g_*Veillonella*, g_*Erysipelatoclostridium*, g_*Actinomyces*	g_*Ruminococcaceae*, g_Lachnospiraceae
Nagata N et al, 2023^129^	Japan	112/112	s_*Ruminococcus torques*	g_*Bifidobacterium*, g_*Dorea*, g_*Roseburia*, g_*Butyricicoccus*
Al-Emran HM et al, 2023^130^	Bangladesh	4/4	s_*Klebsiella pneumoniae*, s_*E. coli* O157:H7, s_*Yersinia pestis*, s_*Porphyromonas*, s_*Enterobacter*	s_*Neisseria meningitidis*, s_*Haemophillus pittmaniae*, s_*Streptococcus parasanguinis*
Mańkowska-Wierzbicka D et al, 2023^131^	Poland	8/14	p_*Verrucomicrobia*, p_*Firmicutes*	p_*Proteobacteria*, p_*Bacteroidetes*, p_*Actinobacteria*
*Long COVID*				
Liu Q et al, 2022^38^^,^∗	China	81/68	s_*Ruminococcus gnavus*, s_*Bacteroides vulgatus*	s_*Collinsella aerofaciens*, s_ *Faecalibacterium prausnitzii*, s_*Blautia obeum*

Note: p_ indicates microbiota at the phylum level, g_ at the genus level, and s_ at the species level. The subjects in Liu's study∗ refer to the ones undergoing COVID infection for at least 6 months.

## IBDs and long COVID

IBDs include Crohn's disease (CD) and ulcerative colitis (UC), majorly in human beings. IBDs are chronic, multifactorial inflammatory disorders of unknown etiology sustained by an exaggerated and poorly controlled immune response against luminal antigens.^50^^,^^51^ As we know, SARS-CoV-2 enters cells by binding to the angiotensin-converting enzyme 2 (ACE2) receptor, which is expressed by numerous cell types, including the small intestine's enterocytes.^52^ Moreover, the inflammatory-related highly activated innate immune cells, including lacked naïve T and B cells, and elevated expression of IFN-β and IFN-λ1, could remain persistently high at 8 months after infection,^53^ which could make the situation of IBD patients worse. The epidemiology, clinical characteristics, and outcomes in acute COVID-19 patients with IBDs have been reported in several studies,^54^^,^^55^ and little data about the prevalence and impact of long COVID in IBD patients were reported recently.^56^, ^57^, ^58^ By recruiting the patients to the hospital to investigate the long COVID and IBDs, Salvatori and colleagues found that 21 out of 53 IBD patients have had SARS-CoV-2 infection, developed long COVID, and asthenia was the most frequent symptom which occurred in nearly two-thirds of studied patients. But no difference in clinical and demographic characteristics was found between patients with and without long COVID, and IBD relapses occurred with the same frequency in the two groups.^56^ Since the abundant expression of ACE2 in the intestine and the persistent GI symptoms in the patients infected with COVID-19, it is reasonable to hypothesize that the SARS-CoV-2 virus may keep in the GI tract for an extended period. Zollner et al reported the expression of SARS-CoV-2 RNA in the gut mucosa around seven months after acute COVID-19 in 32 of 46 IBD patients, and viral nucleocapsid protein persisted in 24 of 46 patients in the gut epithelium. They also found that long COVID was reported in most patients with viral antigen persistence but not in the patients without viral antigen persistence.^57^ This study provides evidence that SARS-CoV-2 virus persistence in the gut can be a basis for immune perturbation in long COVID. Still, the possibility of this persistence underlying the pathophysiology of long COVID needs further clinical trials.

## Potential mechanisms behind GI health and long COVID

The exact mechanism of long CVOID is currently unknown, although some correlations with other diseases or previous health conditions. For example, in 12.7% of patients, the symptoms, such as chest pain, difficulties with breathing, ageusia, or anosmia, and lump(s) in the throat, could substantially increase to at least moderate severity at 90–150 days after COVID-19 diagnosis or matched timepoint.^59^ The authors claimed that this is the first study to report the nature and prevalence of the long COVID condition while correcting for individual symptoms present before COVID-19 and the symptom dynamics in the population without COVID-19 during the pandemic. These findings also highlighted the impact of acute SARS-CoV-1 infection symptoms on the long COVID, such as diarrhea which could cause GI dysfunctions and microbial dysbiosis in the digestive tract during acute COVID-19 might provide a clue for the digest disorders in long COVID. A longitudinal investigation reported that GI long COVID uniquely correlated with the newly expanded cytotoxic CD8^+^ and CD4^+^ T cell populations at T3, including SARS-CoV-2-specific clonotypes, which get activated not during acute disease but at convalescence when long COVID was identified.^60^ All these analyses and investigations provide a resource to understand the heterogeneity and potential contributed biological factors of long COVID and further guide us for mechanisms for studying, monitoring, preventing, and treating long COVID.

According to the current evidence, the GI symptoms of COVID-19 are somehow caused by the direct attack and persistence in the GI tract. As mentioned above, the SARS-CoV-2 enters cells by binding to the angiotensin-converting enzyme 2 (ACE2) receptor, which is highly expressed in the esophagus upper and stratified epithelial cells, absorptive enterocytes from the ileum and colon.^61^ It was also reported to have significantly higher expression in the human and mouse small intestines than in all other organs, including the lungs.^62^ More importantly, TMPRSS2 (transmembrane serine protease 2) and TMRRSS4 (transmembrane serine protease 4), two transmembrane protease serines, can promote SARS-CoV-2 infection of human small intestinal enterocytes and colon.^61^ The SARS-CoV-2 virus activates intestinal receptors, inducing tissue inflammation and causing a high viral load that induces GI problems.^17^^,^^63^ This infection further causes intestinal microbial dysbiosis, which, together with the viral infection, can activate immune cells and provoke the release of pro-inflammatory cytokines. These disturbances could conversely modulate the gut microbiome community and worsen GI symptoms.^64^^,^^65^ Especially in the long COVID patients suffering from consistent GI symptoms, it may be because the virus is persistent in the intestine,^57^ and the microbial dysbiosis and inflammatory conditions keep disturbing the GI tract. This could be supported by Gutiérrez-Castrellón et al, who found probiotic supplementation could have significant effects on reducing symptom duration, viral load, and lung infiltration in acute COVID-19 patients by interacting with the hosts' immune system,^12^ and Wang et al, who indicated the feasibility of alleviating GI symptoms in long COVID patients by way of nutritional modulation of their gut microbiota.^40^

The virus could damage the enterocytes which leads to the dysfunction of intestinal epithelial cells, including disrupted intestinal barrier and increased gut permeability. In the COVID-19 patients with severe illness, there were higher human-origin proteins from both blood and fecal samples, and circulating levels of lipopolysaccharide-binding protein, which suggested gut barrier dysfunction in these patients.^66^ It was also found that severe COVID-19 was associated with high levels of markers (such as tight junction protein zonulin and sCD14) of tight junction permeability and translocation of bacterial and fungal products into the blood.^67^^,^^68^ The higher levels of fungal translocation, which was measured by the fungal cell and wall polysaccharide β-glucan, were found in the plasma of long COVID patients, compared with controls.^69^ More importantly, people with SARS-CoV-2 infection are at increased risk of GI disorders in the post-acute phase of COVID-19.^70^ SARS-CoV-2, like many other viruses, could enhance infectivity by usurping both physiological cell–cell fusion and efferocytosis, disrupting biological barriers.^71^, ^72^, ^73^ For instance, SARS-CoV-2 was found to thrive in infected cells and likely inhibits their clearance and causes inflammation and barrier dysfunction.^74^ It is reported the important role of angiotensin II in SARS-CoV-2 infections for the COVID-19 up-regulated ANG II could disrupt efferocytosis inducing endothelial cell senescence and vascular barrier dysfunction via angiotensin II type 1 receptors.^72^^,^^75^ Taken together, disrupted barrier functions are related to the observed pathophysiological changes in long COVID.

## Potential treatment on GI tract for long COVID

As discussed, significant GI sequelae have been reported in long COVID with GI functional disorders and microbial dysbiosis.^36^^,^^40^^,^^63^ This altered gut microbiome may enrich the opportunistic infectious organisms, deplete beneficial commensals in the intestine, and alter the course of respiratory infections, including COVID-19, through the gut–lung axis, which has been recognized previously in influenza and other respiratory infections^76^, ^77^, ^78^ and discussed in the recent papers.^17^^,^^79^, ^80^, ^81^ Therefore, the treatment by retrieving the gut microbial community, such as probiotics and beneficial-organism-related products, could be used to improve GI conditions and enhance barrier functions in long COVID. Probiotic supplementation has been reported that it could significantly reduce symptom duration, viral load, and lung infiltration in acute COVID-19 patients by interacting with the hosts' immune system.^12^ The GI symptoms in long COVID patient was reported to be alleviated by high-fiber formula nutritional modulation through remodeling gut microbiota profiles.^40^ *Faecalibacterium prausnitzii*, a butyrate-producing anaerobe typically associated with good health, has been reported reversely correlated with disease severity in acute COVID-19 ^82^. Butyrate, a molecule found to benefit many diseases,^32^^,^^83^^,^^84^ may be considered one of the potential treatments for long COVID.

Fecal microbiota transplantation (FMT) is used for several diseases or disorders, such as clostridium difficile infection (CDI),^85^ metabolic disorders,^86^ hepatic encephalopathy,^87^ and Good's syndrome.^88^ Given gut microbial dysbiosis, effects of the gut–lung axis, and associations between gut microbiota and long COVID, FMT might be practical in long COVID patients. However, to our knowledge, there has not been any completed clinical trial on the safety and efficacy of FMT in patients with COVID-19 or long COVID. On one hand, the clinical trial NCT04251767, which conducted a randomized-controlled trial on acute COVID-19 patients to discover the effectiveness of washed microbiota transplantation on improving the disease severity, was withdrawn to follow new disciplines of the government.^89^ On the other hand, the ongoing COVID-19 pandemic has implicated the FMT in treating CDI and other diseases due to the unknown impacts of SARS-CoV-2 on the safety and efficacy of FMT.^90^^,^^91^

## Challenges and future directions

Long COVID is a collective term for reports from various parts of the world showing that a significant proportion of people who recovered from COVID-19 suffer from various health issues. However, it is a collection of at least four distinct clinical entities, including post-intensive care syndrome, post-viral fatigue syndrome, permanent organ damage, and long-term COVID-19 syndrome.^92^ Therefore, one of the significant challenges for long COVID is the diagnosis. Even though researchers proposed some clinical criteria,^92^ the high mild or asymptomatic infections of SARS-CoV-2 (80%)^93^ make the diagnostics complicated. The fact that nearly all symptoms reported in children and adolescents with long COVID are also reported in similar frequencies in those without evidence of infection further highlights the challenge of distinguishing long COVID-associated symptoms from pandemic-related symptoms and other diseases.^94^

The COVID-19 pandemic is changing the management of many diseases and treatments. For instance, the patients who need FMT which has been dramatically implicated due to the unknown impacts of SARS-CoV-2 on FMT safety and efficacy.^90^^,^^91^ Also, the IBD patients whose routine endoscopy performance was suspended^95^ may lead to more severe results, such as colon cancer. Therefore, during the post-COVID era, gastroenterologists need to continue offering urgent endoscopic investigations and other relevant investigations to patients with IBDs and other intestinal diseases requiring endoscopy to avoid chronic disease worsening.

Omics represent the collective technologies that help to investigate the roles of biological molecules and organism cell actions which include genomics,^42^ transcriptomics (mRNA), proteomics (proteins), and metabolomics (metabolites). These techniques have been widely used in vaccine development and drug repurposing,^96^ significantly assisting in describing gene/protein expression profiles and their complex effects on the SARS-CoV-2 virus.^97^, ^98^, ^99^ There has been some work on integrated multi-omics signatures, such as meta-transcriptome sequencing,^100^ proteomic and metabolomic analyses in COVID-19 patients,^101^ and trans-omics characteristics among patients with different disease severity.^102^ Since the asymptomatic of the new SARS-CoV-2 variants and joint development of long COVID in the post-COVID era, the multi-omics analysis could help us gain insight into this disease, understand crosstalks among organs (*e.g.*, gut–lung axis, gut–brain axis), and contribute to our understanding of the underlying pathogenesis of long COVID and potential therapeutic strategies for the suffering patients.

During the pandemic, some populations are being hit especially hard by COVID-19 because of disparities in health care, social conditions, structural racism, and other factors. For instance, nearly 20% of U.S. counties are disproportionately black, accounting for 52% of COVID-19 diagnoses and 58% of COVID-19 deaths nationally.^103^ More specifically, black women with high preexisting conditions and overrepresented in occupations where exposure to the virus are at times as likely to die of COVID as white men.^104^^,^^105^ As one in five people infected with the SARS-CoV-2 virus will develop chronic symptoms,^106^ *i.e.*, long COVID, it is essential to understand the extent to which it burdens people of color and other underrepresented groups or populations. There are unknown factors of COVID on the infant microbiome and their long-term development.^81^^,^^107^

It is estimated that about 65 million people have long COVID worldwide if we conservatively estimated an incidence of 10% of over 651 million documented COVID-19 cases.^59^ Given the broad range of effects of long COVID on various organs and systems, the pathogenesis of long COVID is complex and multifactorial.^108^ Current pathogenic understanding of long COVID is in its infancy, although it was suggested that significant determinants of the persistence of COVID-19 reactivation of other viruses, autoimmunity, and uncontrolled inflammation. But a better understanding of the long COVID spectrum and underlying mechanisms may pave the way to better prevention and therapeutic strategies.

## Conflict of interests

Jun Sun and Yinglin Xia are the member of *Genes & Diseases* Editorial Board. To minimize bias, they were excluded from all editorial decision-making related to the acceptance of this article for publication. The remaining authors declare no conflict of interests.

## Funding

This project was supported by the IllNET RECOVER pilot grant (to J.S. and Y.X.), 10.13039/100011684Crohn's & Colitis Foundation Senior Research Award (No. 902766 to J.S.), United States Department of Defense 10.13039/100000090Congressionally Directed Medical Research Programs (No. BC191198 to J.S.), and 10.13039/100000738VA Merit Award BX-19-00 to J.S. The contents do not represent the views of the United States Department of Veterans Affairs or the United States Government. The study sponsor plays no role in the study design and data collection, analysis, and interpretation.
